# Valid and accurate simple equation to predict 3,000-m steeplechase performance

**DOI:** 10.3389/fspor.2024.1402792

**Published:** 2024-07-24

**Authors:** Alexis Barbry, Lucie Lerebourg, Ghazi Racil, Mohamed Chedly Jlid, Jérémy Coquart

**Affiliations:** ^1^ Centre d’Études des Transformations des Activités Physiques et Sportives, Normandie University, UNIROUEN, Rouen, France; ^2^ Univ. Lille, Univ. Artois, Univ. Littoral Côte d'Opale, ULR 7369 - URePSSS - Unité de Recherche Pluridisciplinaire Sport Santé Société, Lille, France; ^3^ LAMHESS Lab, Université Côte d’Azur, Nice, France; ^4^ Orthodynamica Center, Mathilde Hospital 2, Rouen, France; ^5^ Higher Institute of Sport ansd Physical Education of Ksar Saïd, University of Manouba, Tunis, Tunisia

**Keywords:** validity, running, hurdling, athlete, athletics

## Abstract

**Introduction:**

Predict running performances is very important for athletes and trainers. Sport researchers have therefore developed certain tools to predict running performances, but only in non-obstacle races. This study aimed to develop and test the validity and accuracy of an equation for predicting 3,000-m steeplechase performance (*Perf_Steeple_*).

**Methods:**

The official rankings of French runners for the 3,000-m track-running (*Perf_3000_*) and 3,000-m steeplechase events were examined. Age, height and body mass were collected. From 146 included athletes, two groups were randomly composed: one comprising 80% of the sample (*n* = 117) to develop a simple equation to predict *Perf_Steeple_* (*i.e.*, development group) and the other comprising the remaining 20% (*n* = 29) to test the validity and accuracy of the developed prediction equation (*i.e.*, cross-validation group).

**Results:**

The simple prediction equation included *Perf_3000_* and age: $Perf_{Steeple}=-57,165+1,147\times Perf_{3000}+0,955\times age$. No significant difference was noted between the actual and predicted performances. Predicted performances were significantly correlated with the actual ones, with a very high correlation coefficient (*p *< 0.001; *r* = 0.929). Bias and 95% limits of agreement were −5 ± 24 s, *i.e.*, −0.8 ± 7.6%. In 95 of 100 new predictions, the difference between actual and predicted performance would be less or equal to—5 ± 24 s.

**Discussion:**

The study confirms the validity and accuracy of the equation for predicting *Perf_Steeple_*. Predictions using this simple equation may be used in training and competitions for athletes and coaches. *Perf_Steeple_* = −57,165 + 1,147 X *Perf_3000_* + 0,955 X *age*.

## Introduction

The prediction of running performances is very important for athletes and trainers (1–3). Predictions might be used to detect talents, select a speed for specific pace training sessions, and even evaluate the effect of a training program (1–4). Sports researchers have therefore long sought ways to accurately predict running performances. Several authors have developed certain methods and tools (*e.g.,* power law, critical speed or nomograms) (5–8). These different tools are used to predict middle- and long-distance track-running performances, but only in non-obstacle races. Consequently, the studies on predicting the track-running performances of the steeplechase remain very limited.

The 3,000-m steeplechase is a technical middle-distance running event with the athletes crossing 28 barriers and 7 barriers followed by a water pit (water-jumps). The main technical/regulatory difference between the sexes is the height of the barriers (*i.e.,* 0.914 m and 0.762 m for men and women, respectively).

Some predictive variables of a 3,000-m steeplechase might be the same variables as for flat middle- and long-distance running. Maximal oxygen uptake ($\dot{V}$O_2max_), endurance capacity or running economy are key physiological factors of running performances (9). These factors known to be dependent on age, sex, height, body mass and/or body mass index (BMI) (10, 11). For age, few studies have investigated it relationship with performance among steeplechase runners. One study has nevertheless shown that the age of elite athletes at peak performance on the 3,000-m steeplechase is around 25–27 years old (12). Sex also appears to be a variable to consider in predicting performance in the 3,000-m steeplechase. Differences in the technique used to cross the steeplechase barriers have been identified between the sexes (13), probably due to the lower barrier height for women. Therefore, the same equation cannot be used for men and women. These differences in hurdling technique may also be due to the approach velocity and body height (14). Sánchez Muñoz et al. (15) showed that runners of long-distance events (including the 3,000-m steeplechase) are taller than runners of shorter distances (800-m and 1,500-m). Being taller is often associated with longer legs, which would facilitate barrier crossing. Moreover, it is well established that a lower BMI positively impacts running performances (16). Consequently, due to the technical aspect and the energy cost of crossing barriers (17), height, body mass and BMI appear to be other predictive variables of steeplechase performance.

The purpose of the current study was to develop and test the validity and accuracy of a simple equation in predicting 3,000-m steeplechase performance (*Perf_Steeple_*) from individual variables (*e.g.*, age, height, body mass and BMI) in men.

## Methods

### Subjects

All official French rankings from the French athletics federation (FFA for *Fédération Française d'Athlétisme*) in 2019 for the 3,000-m track (*n* = 5,385) and 3,000-m steeplechase (*n* = 1,333) events were retrospectively scrutinized. From these rankings, all adult athletes (≥18 years) who had participated in the two races were retained (*n* = 387). Runners who had not self-reported their body mass and/or height were then removed from the analysis (*n* = 211). Thus, 176 athletes were included in this stage. Because of the small sample of women (*n* = 25) and to avoid possible sex bias (different height of the barriers between men and women), only men were included (*n* = 151). Runners who had maintained a higher speed in the 3,000-m steeplechase than in the 3,000-m race were also eliminated (*n* = 5).

### Design

From the 146 remaining male athletes, two groups were randomly composed using statistical package for the social sciences (SPSS) software. One comprising 80% of the sample (*n* = 117) to develop a simple prediction equation (*i.e.*, development group) and a second comprising the remaining 20% (*n* = 29) to test the validity and accuracy of the developed simple prediction equation (*i.e.*, cross-validation group). To prevent problems with the normality of the distribution, approximately 30 runners were included in the cross-validation group (18, 19).

### Methodology

For each athlete, the birth date (to calculate the age), height and body mass (to calculate the BMI), as well as the race times on the *Perf_Steeple_* and 3,000-m race (*i.e.*, *Perf_3000_*) were recorded. This study was approved by the national ethics committee for research in sports sciences **(**CERSTAPS 2019-22-02-31). The protocol for this study was legally declared, in accordance with the European general data protection regulations.

### Statistical analysis

Standard statistical methods were used to calculate the means and standard deviations (SD). The normal Gaussian distribution was veriﬁed by the Shapiro-Wilk test.

To investigate whether an equation could be developed to predict *Perf_Steeple_*, a Pearson product moment correlation was used to evaluate the association between the dependent (*i.e.*, *Perf_Steeple_*) and several independent variables (*i.e.*, *Perf_3000_*, age, height, body mass and BMI).

A simple prediction equation was then developed from the significantly correlated variables using stepwise multiple linear regression analysis.

The variance inflation factor (VIF) was used to detect the severity of multicollinearity among the independent variables in the regression model. Multicollinearity was considered very low when VIF < 5 (20, 21).

Fisher's tests were used to examine the contribution of each variable to the model, and confirmation was based on the analysis of standardized *β* coefficients.

The relationship between *Perf_Steeple_* estimated by the simple prediction equation and actual *Perf_Steeple_* was analyzed using the Bravais-Pearson method and quantified using Pearson's correlation coefficient. The autocorrelation in the residuals was examined with the Durbin-Watson test.

Student's paired samples *t*-test was used to compare the actual and predicted *Perf_Steeple_*. The magnitude of the differences was assessed by the effect size (ES) using cohen's *d* (22). ES was considered as small (*d* = 0.20), medium (*d* = 0.50) or large (*d* = 0.80) (22).

The association between the actual and predicted *Perf_Steeple_* was tested using the Bravais-Pearson method and quantified by Pearson's correlation coefficient. It was considered a correlation of *r* = 0,90 or more as very high, between 0,70 and 0,89 as high, between 0,50 and 0,69 as moderate, and between 0,26 and 0,49 as low (23, 24).

The bias (*i.e.*, difference between actual and predicted *Perf_Steeple_*) and 95% limits of agreement (95% LoA, *i.e.*, ± 1.96 SD) were computed according to the Bland-Altman method (25).

Statistical significance was set at *p* < 0.05 and all analyses were performed with the SPSS (release 20.0, Chicago, IL, USA).

This statistical method was realised by several authors to predict running performances (20, 24, 26).

## Results

The characteristics of the athletes in both groups are presented in Table 1.

**Table 1:** Descriptive data and variables used to develop the simple equation of prediction (mean values ± *SD*).

	Group for the development of the prediction equation	Group for the cross-validation of the prediction equation
Sample size	117	29
Age (y)	28.4 ± 8.3	27.8 ± 7.8
Body mass (kg)	62.9 ± 7.3	63.4 ± 6.3
Height (m)	1.77 ± 0.07	1.78 ± 0.05
Body mass index (kg.m^−2^)	20.0 ± 1.6	20.1 ± 1.5
*Perf_Steeple_* (min and s)	10 min 21 s ± 1 min 03 s	10 min 20 s ± 1 min 03 s
*Perf_3000_*_m_ (min and s)	9 min 28 s ± 47 s	9 min 23 s ± 45 s

Significant bivariate correlations were found between *Perf_3000_* (*p* < 0.001, *r* = 0.896), *age* (*p* < 0.001, *r* = 0.474), body mass (*p* = 0.049, *r* = 0.154), BMI (*p* = 0.001, *r* = 0.279) and *Perf_Steeple_*.

Stepwise multiple linear regression analysis using *Perf_3000_* and *age* as potential independent variables yielded the simple prediction equation.

This equation was as follows:

$$Perf_{Steeple}=-57,165+1,147\times Perf_{3000}+0,955\times age$$

*Perf_Steeple _= *−57,165 + 1,147 ×

*Perf_Steeple _*= −57,165 + 1,147 ×  *Perf_3000_* + 0,955 × age

with *Perf_Steeple_* and *Perf_3000_* in seconds, and *age* in years.

VIF was 1.206 for both *Perf_3000_* and age.

The increases in *r^2^* by adding a second predictor (*i.e.*, age) into the prediction equation were significant with F(1,114) = 8.083 (*p* = 0.005). Moreover, Fisher's test revealed a *p* < 0.05.

The performance estimated by the simple prediction equation (including the 2 independent variables: *Perf_3000_* and *age*) was significantly correlated with the actual *Perf_Steeple_* (*r* = 0.903 and *r^2^* = 0.8162).

The standardized *β* coefficients and *p*-values on the Student's *t*-test were 0.844 (*p* < 0.001), and 0.126 (*p* = 0.005) for *Perf_3000_* and *age*, respectively.

The Durbin-Watson test indicated no autocorrelation in the residuals, with a value of 1.725. The standardized residuals followed a straight line, showing that no residual was too high, so the prediction was valid for all performances.

No significant difference was noted between the actual and predicted *Perf_Steeple_* (*p* = 0.263), and the magnitude of the difference was considered trivial (ES = –.08) (22).

**Figure 1:**
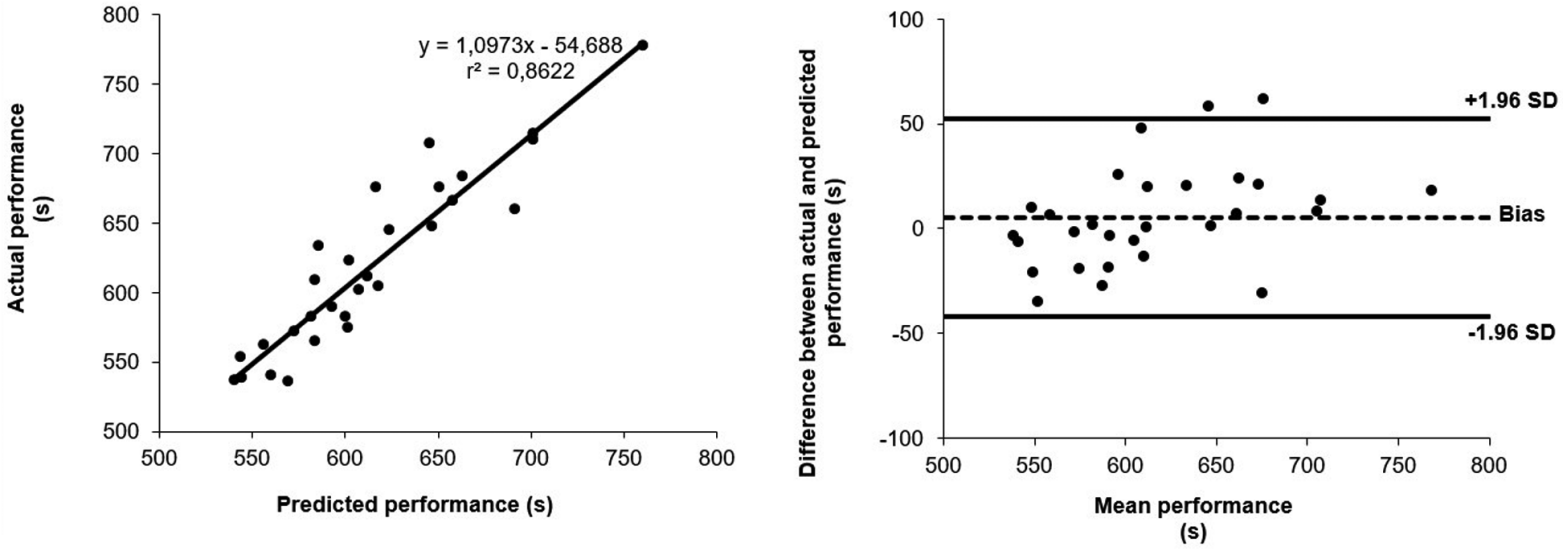
Top panel: association between actual and predicted performance from a simple equation to predict the 3,000-m steeplechase event. The solid line is the linear regression. r2 is the coefficient of determination. Bottom panel: Bland and Altman plots for the comparison between actual and predicted performance in the 3,000-m steeplechase event. Dashed line is the bias, solid lines are the 95% limits of agreement (25).

Predicted *Perf_Steeple_* was significantly correlated with actual *Perf_Steeple_* (*p* < 0.001). A very high correlation coefficient was found according to Munro's scale (23) (*r* = 0.929, Figure 1).

The bias and 95% LoA were −5 ± 24 s, *i.e.*, −0.8 ± 7.6% of mean *Perf_Steeple_* (Figure 1). In 95 of 100 new predictions, the difference between actual and predicted performance would be less or equal to −5 ± 24 s.

## Discussion

The purpose of this study was to develop and test the validity and accuracy of a simple equation to predict *Perf_Steeple_*. The main result suggests that a simple equation (*i.e.*, *Perf_Steeple_* = −57.165 + 1.147 × *Perf_3000_* + 0.955 × *age*) is a valid and accurate method for predicting *Perf_Steeple_*. Although no study has focused on predicting *Perf_Steeple_* from a simple equation, a few studies have validated predictive methods for the flat 3,000-m with results comparable (*i.e.*, from low bias and 95% LoA) to our own (3, 27). This confirms the validity of the present simple equation for the 3,000-m steeplechase. This simple equation can complement other methods/tools. A long-distance runner (who wanted to predict *Perf_Steeple_* without reference in *Perf_3000_*) could use the methods and tools available in the literature to firstly estimate *Perf_3000_* (5–8). Next, use this estimated *Perf_3000_* to predict his *Perf_Steeple_* with the simple equation.

The present results suggest that the main predictor of *Perf_Steeple_* is *Perf_3000_*. The duration in these two events is relatively close (*i.e.,* 8%–10% prolonged effort in the 3,000-m steeplechase, having little impact on the importance of the $\dot{V}$O_2max_) (17, 28, 29). The 3,000-m steeplechase generally produces a deceleration during the takeoff step for both hurdling and water-jumps (30). In addition to requiring a high level of $\dot{V}$O_2max_ (29), *Perf_Steeple_* requires a high technical ability to cross the barriers. This high technical ability (*e.g.,* more efficiency over barriers) might be developed by plyometric training (17). The inclusion of plyometric training could support runners to be more efficient over barriers (17). These types of exercises increased muscular fitness which can be useful to decelerating, landing and exiting steeplechase barriers (31).

Based on the statistical method used (20, 24, 26), this study shows that *age* contributes to performance prediction in the *Perf_Steeple_*. Its inclusion provides only a small gain in accuracy. It can be explained by the observation that most steeplechasers are between 20 and 35 years old (Table 1). This is not surprising, given that a peak age of around 25 years old has been noted for men (12). However, the inclusion of *age* could be explained by the age-related physiological modifications with adult aging (32). A progressive reduction in $\dot{V}$O_2max_ appears to be the primary mechanism associated with performance declines (33).

The current study suggests that height, body mass and BMI are not predictive variables of *Perf_Steeple_*. This result might be explained by the inclusion of *Perf_3000_* in the equation. Although height is slightly associated with speed in running events, body mass and BMI seems to be a better indicator of *Perf_3000_* (16). The impact of height, body mass and BMI on *Perf_3000_* would explain why these variables were not included in the equation (*i.e.,* they are already included in *Perf_3000_* and would therefore be redundant).

The main limitation of this study is that the running performances were not executed in similar conditions (*i.e.,* environmental, physical and psychological). Another limitation was the self-declaration of body height and mass and thus the calculation of the BMI. However, runners are known to self-report accurately for this type of data (34). Finally, this equation is a valid and accurate method only in male athletes. The height of the barriers with a 0.152-m difference between sexes, can lead to differences in the running-hurdling (13). The lower barrier height relative to body height for women might help them to maintain race pace when crossing the barriers (14). However, they are more affected by the water-jump (*e.g.,* lower approach velocity, shorter jump) than those of the men (13). This is probably because they need a longer stride to cross the water due to their generally shorter leg length. Thus, a future perspective might be to develop a specific prediction equation to predict *Perf_Steeple_* for women.

## Conclusion

The results of the present study indicate the validity and accuracy of a simple equation to predict *Perf_Steeple_*. This tool could be useful for athletes and coaches to predict a 3,000-m steeplechase. It can enable them to adapt their training and to select the optimum speed for the athlete's performance. It can be use for national directors in charge of performance in athletics federations to detect talent in the 3,000-m steeplechase and thus orient young athletes more easily.

## Data Availability

The raw data supporting the conclusions of this article will be made available by the authors, without undue reservation.
